# Investigation of Chlorhexidine and Chitosan Gel-Based Coatings for the Prevention of Intravascular Catheter-Associated Infections Following Quality by Design Approach

**DOI:** 10.3390/biomedicines12092032

**Published:** 2024-09-05

**Authors:** S P Yamini Kanti, Mahwash Mukhtar, Martin Cseh, László Orosz, Katalin Burián, Rita Ambrus, Orsolya Jójárt-Laczkovich, Ildikó Csóka

**Affiliations:** 1 Institute of Pharmaceutical Technology and Regulatory Affairs, University of Szeged, 6720 Szeged, Hungary; mahwash.mukhtar@gmail.com (M.M.); ambrus.rita@szte.hu (R.A.); csoka.ildiko@szte.hu (I.C.); 2 Center of Excellence for Interdisciplinary Research, Development and Innovation, 3D Centre University of Szeged, 6722 Szeged, Hungary; cseh.martin@szte.hu; 3 Department of Medical Microbiology, Albert Szent-Györgyi Medical School, University of Szeged, 6720 Szeged, Hungary; orosz.laszlo@med.u-szeged.hu (L.O.); burian.katalin@med.u-szeged.hu (K.B.)

**Keywords:** antibiofilm, catheter-associated infection, chlorhexidine, polymeric coating, quality by design

## Abstract

Intravascular catheter-associated infections pose a significant threat to the health of patients because of biofilm formation. Hence, it is imperative to exploit cost-effective approaches to improve patient compliance. With this aim, our present study reported the potential of an antimicrobial polymeric gel coating of chitosan (CS) and chlorhexidine (CHX) on the marketed urinary catheters to minimize the risk of biofilm formation. The study involved the implementation of the Quality by Design (QbD) approach by identifying the critical parameters that can affect the coating of the catheter’s surface in any possible way. Later, design of experiments (DoE) analysis affirmed the lack of linearity in the model for the studied responses in a holistic manner. Moreover, in vitro studies were conducted for the evaluation of various parameters followed by the antibiofilm study. The coating exhibited promising release of CHX in the artificial urinary media together with retention of the coating on the catheter’s surface. Therefore, this study aims to emphasize the importance of a systematic and quality-focused approach by contributing to the development of a safe, effective, and reliable catheter coating to enhance intravascular catheter safety.

## 1. Introduction

Catheter-associated urinary tract infections (CAUTIs) are the most common nosocomial infections, posing a significant healthcare challenge by affecting millions of individuals annually [1]. The causative agents associated with CAUTIs are *Escherichia coli*, *Klebsiella pneumoniae*, *Proteus mirabilis*, and *Enterococcus faecalis* [2,3]. The infection arises when urinary catheters, commonly used in clinical settings for patients with impaired bladder functions, undergoing childbirth or surgical procedures, become a channel for microbial colonization. The prolonged use of a catheter provides an environment for bacterial adherence and biofilm formation on the catheter’s surface resulting in infections. These infections can lead to other diseases and increase mortality in patients, especially in the immune-compromised population. CAUTIs cause symptoms such as fever, pain, and discomfort; leading to serious complications such as sepsis and kidney damage [4]. In addition to the impact on patient health, CAUTIs can also burden the patient emotionally and economically because of long hospital stays and cost of treatment, respectively. Therefore, preventing and treating CAUTIs is an important priority in healthcare settings, emphasizing the need for innovative approaches such as effective antimicrobial coatings for urinary catheters as explored in this study, thereby improving therapeutic outcomes and patient compliance [1].

The importance of antimicrobial coatings for catheters lies in their ability to prevent the formation of bacterial biofilm associated with the use of catheters for a prolonged duration. For this purpose, catheters can be coated with different bactericidal or bacteriostatic antimicrobial agents such as silver [5,6], antibiotics [7,8,9], enzymes [10], nitric oxide [11,12], polymers [13], etc., which can act as a protective barrier, inhibiting microbial adherence and biofilm formation [14]. The studies conducted using these antimicrobial coatings have shown reduction and prevention in the incidence of bacterial colonization and biofilm formation on the catheter’s surface, therefore reducing the risk of infection and the associated complications.

Moreover, the outcome can be further enhanced using an intravesical catheter together with drug delivery directly to the bladder or site of action to facilitate the reduction in bacterial count. Intravesical drug delivery plays an important role in the prevention of bacterial biofilms thus reducing the risk of CAUTIs [15]. Therefore, the development of antimicrobial film-coated catheters for intravesical drug delivery can be a rational strategy for maintaining the effective drug concentration at the target site to perform its pharmacological activity as long as the catheter is inside the body, consequently reducing the rate of incidence of CAUTIs [16].

It is, however, central to select the appropriate coating agent and drug while developing a gel coating on the catheter’s surface for intravascular delivery. The most widely explored antibacterial agents in coatings are nitrofurazone, norfloxacin, ciprofloxacin, triclosan, and chlorhexidine (CHX). In this work, CHX was selected as the drug of choice. According to the literature, CHX, a quaternary ammonium antiseptic, is in wide use because of its broad spectrum of antimicrobial and antibacterial properties [17]. CHX exhibits bacteriostatic activity at lower concentration and bactericidal activity at higher concentrations. However, the effectiveness varies with the different bacterial species [18]. It acts by disrupting the microbial cell membrane, resulting in cell lysis and death. It is widely used in clinical and healthcare environments and is often incorporated into oral mouthwashes to reduce plaque, gingivitis, and oral bacterial exposure [19]. In addition, to oral use, CHX is formulated into aqueous and alcoholic antiseptic solutions to be used by hospitals. It is also widely used to clean and disinfect the wounds, cuts, or abrasions to promote healing and prevent inflammation. Chlorhexidine also plays an important role in preventing and treating urinary tract infections, especially catheter-associated UTIs. When used as a coating for indwelling urinary catheters, CHX effectively prevents bacterial colonization and can also eradicate the already existing biofilms as well as the adjuvant biofilms without developing bacterial resistance as compared to other antibacterials [20,21]. Previous research has also reported that CHX is biocompatible when used as a coating on urinary catheters through hemocompatibility and skin testing, as it showed minimal hemolysis, no signs of dermal toxicity, and stable drug release confirming its safety and suitability for medical applications [22]. Moreover, the incidence of reaction to CHX is very rare [14,20,23]. For coating, it is essential to select a biocompatible polymer with the possibility of reducing bacterial adhesion on its surface. Keeping this in mind, chitosan (CS) was selected as a bacteriostatic and bactericidal polymer. Chitosan is a polycationic deacetylated polysaccharide obtained by partial N-deacetylation of chitin, a natural biopolymer derived from crustacean shells, shrimps, or other micro-organisms such as fungi. It is a linear polysaccharide with β-(1→4)-linked 2-acetamido-2-deoxy-β-D-glucopyranose and 2-amino-2-deoxy-β-D-glucopyranose. Because of unique properties such as biocompatibility, bio-degradability, non-toxicity, and low cost, CS is a potential candidate to be used for the development of coatings [24,25]. 

In the development of new pharmaceutical formulations, quality, safety, and efficacy can be ensured with the Quality by Design (QbD) approach throughout the lifecycle of the product. It is a systematic approach to drug product development that focuses on designing and developing quality from product conceptualization to commercialization. Hence, it provides a structured framework to define the quality target product profile (QTPP) by scrutinizing the critical quality attributes (CQAs), critical material attributes (CMAs), and critical process parameters (CPPs) [26]. The International Council for Harmonization (ICH) also laid down some guidelines as quality tools, such as Q9 (Quality Risk Management) [27]. The goal is to develop a product that consistently meets the required quality standards and regulatory requirements. The process involves several steps, including risk assessment, followed by the optimization of critical factors through the design of experiments (DoE) holistic method. This proactive approach facilitates the optimization of formulation confirming the enhanced product performance with minimized risk of defects or failures [28,29]. In this study, we used a systematic approach to develop and investigate the potential of chlorhexidine and chitosan gel-based coatings for the prevention and management of catheter-associated urinary tract infections. This study focused on developing a coating that reduces bacterial adhesion and biofilm formation on the surface of urinary catheters, thereby minimizing the risk of CAUTIs, by utilizing the broad-spectrum antimicrobial properties of chlorhexidine and the biocompatibility of chitosan. Our research highlights the importance of a systematic and quality-driven approach for the development of safe, effective, and reliable coating through a Quality by Design (QbD) approach.

## 2. Materials and Methods

### 2.1. Materials

Chlorhexidine digluconate 20% *w*/*v*, glacial acetic, sodium chloride, urea, and potassium chloride were kindly provided from Molar Chemicals Ltd. (Halásztelek, Árpád u. 1, Budapest, Hungary). CS [75–85% deacetylated low molecular weight, 50–190 kDa, Poly(D-glucosamine)] and Creatinine anhydrous ≥ 98% were from Sigma Aldrich (3300 S 2nd St #3306, St. Louis, MO, USA). Polylactic acid (PLA) white filament under the brand name Sunlu was provided by Variometrum Kft. (Szállás u. 17, 1107, Budapest, Hungary). Foley Ballon Catheter Silicon Coated 14CH size was provided from Renault Petersen Limited, (5 Bankside, Hanborough Business Park Long Hanborough, Witney, Oxfordshire, OX29 8LJ, United Kingdom). Sodium Phosphate was from Spectrum Chemical Mfg. Corp. (769 Jersey Ave, New Brunswick, NJ, United States). Bovine Serum Albumin Lyophilized powder purity ≥ 97% was from Biosera (Kenderike u.1. Budapest, Hungary).

### 2.2. Bacterial Strains 

The following strains were used in our experiments: *Escherichia coli* ATCC 25,922 (*E. coli*), *Staphylococcus aureus* ATCC 25,423 (*S. aureus*, an MRSA strain), and *Pseudomonas aeruginosa* ATCC 27,853 (*P. aeruginosa*).

### 2.3. Quality by Design

**Figure 1:**
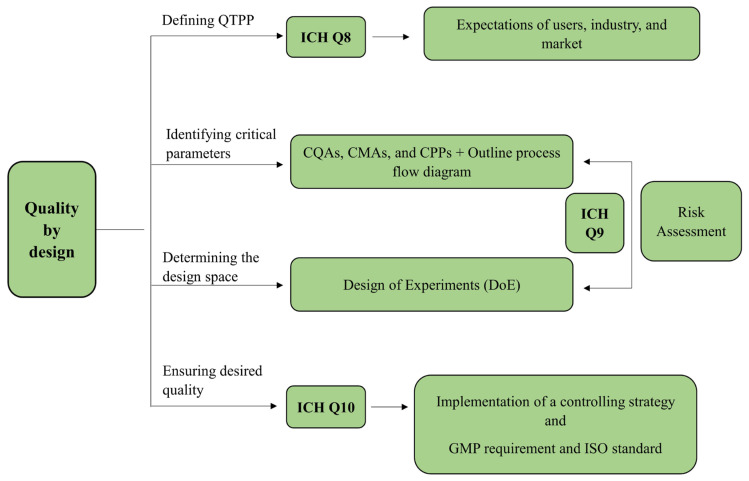
Schematic representation of implementation of QbD design [30,31].

QbD is a systematic approach to drug product development that focuses on designing and developing quality in the product. The structured framework of QbD helps in identifying the critical parameters such as QTPP, CQAs, CMAs, and CPPs, to develop a product that consistently meets the required quality standards and regulatory requirements. Figure 1 represents the schematic process of QbD implementation.

#### 2.3.1. Knowledge Space Development and Defining QTPP

Collection of previously published literature and scientific data in an organized systematic way encompasses the term knowledge space development. After thorough study, evaluation, and scrutiny, the first step is to define the QTPP based on the considerations of patients, industries, and healthcare staff such as clinicians. The guidelines for defining QTPP have been described in ICH Q8 including all the requirements related to quality, safety, and efficacy depending on the dosage form, strength, route of administration, site of action, etc. [32].

#### 2.3.2. Identification of CQAs, CMAs, and CPPs 

After identifying QTPP, the next step is to identify CQAs which ensures that quality is achieved in the final product. CQAs are the physical, chemical, biological, and morphological attributes within an appropriate range to ensure the desired quality of the product. Furthermore, the CQAs are also influenced by the therapeutic aims and predefined outcomes or goals. The next step is to identify the CMAs and CPPs which are the critical characteristics of the material and production process, respectively, that may impact CQAs. Together, these include properties of the drug, excipient, and the process variables [33].

#### 2.3.3. Risk Assessment

Risk assessment helps in identifying critical factors affecting the development of a drug delivery system while minimizing the associated risks and improving the overall product quality. In the case of urinary catheter coatings, this approach facilitated scrutiny of critical factors at all stages to address the risks associated with the product development. Through careful selection of an appropriate antimicrobial agent, polymer, concentration of active ingredient, type of solvent, etc., the process of coating can be optimized to provide maximum protection against CAUTIs while minimizing complications [34]. After intensive evaluation and selection of risk factors, the interdependence between QTPP and CQAs, and between CPPs/CMAs and CQAs is assessed using three-level grading of Low (L), Medium (M), and High (H). For this purpose, Lean QBD software^®^ Version 1.4.3 (QBD Works LLC, Fremont, CA, USA) is employed. The critical attributes are graded by the software in terms of their estimation of occurrence. The obtained outcomes are generated as Pareto charts which helps in further optimizing these critical parameters. 

### 2.4. Design of Experiments Using Box–Behnken (BB) Design Method

DoE is a robust and efficient method for the optimization of the development process of pharmaceutical drug delivery systems and devices. It ensures the quality of the developed system through the identification and optimization of critical factors that were determined during the initial risk assessment. DoE allows studying multiple factors and their interactions, helping to understand all the possible levels of interference throughout the process. Traditional methods of product development are time consuming and cumbersome, whereas the DoE approach is cost-effective and an efficient time-saving method for assessment [26]. In this study, we have used BB design to investigate the relationship between parameters and their impact on dependent variables with the minimal number of experimental runs generated by STATISTICA^®^ 12 software [35]. (Table S1 Supplementary Files).

The independent variables chosen through risk assessment were the concentration of CS (X_1_, lower limit: 1.5% *w*/*v*, upper limit: 2.5% *w*/*v*), pH (X_2_, lower limit: 4.8, upper limit: 5.2), and temperature (°C) (X_3_, lower limit: 20 °C, upper limit: 40 °C). On the other hand, the dependent variables selected were Y_1_: polarity, Y_2_: retention of coating, and Y_3_: % CHX release from coated catheters. ANOVA was applied in the software for statistical significance (*p* < 0.05). The polynomial model (Equation (1)) was obtained after the regression analysis of each response, which is as follows:

Y = β_0_ + β_1×1_ + β_2_X_2_ + β_3_X_3_ + β_12_X_1_X_2_ + β_13_X_1_X_3_ + β_23_X_2_X_3_ + β_11_X_12_ + β_22_X_22_ + β_33_X_32_ (1)

where Y is the response, X_1_–X_3_ are the main effects of factors, and X_1_X_2_, X_1_X_3_, and X_2_X_3_ are the interaction factors. β_0_ is the constant and β_1_–β_3_ are the coefficients of factors.

### 2.5. Preparation of Catheter Samples and Coating

In our study, Foley catheter fragments are prepared and coated with a chlorhexidine-chitosan based film to enhance their surface properties. Various concentration of chitosan are utilized under controlled ph and temperature, with the coating process carried out using dip coating process. The aim is to achieve a uniform and stable coating on the catheter surface, optimizing their performance for intended purpose. Table 1 represents the detailed process of coating.

### 2.6. Weight-Gain Analysis of Coated Catheters 

All the catheter pieces were weighed before the coating and, subsequently, after the coating to ensure that the coating had adhered to the surface of catheters. The weight of the coated catheter pieces was subtracted from the initial weight of the uncoated catheter pieces to acquire the required results.

### 2.7. Morphological Analysis Using Scanning Electron Microscopy (SEM) 

SEM micrographs of the coated and uncoated catheter pieces were obtained by using scanning electron microscopy (SEM) (Hitachi S4700, Hitachi Scientific Ltd., Tokyo, Japan) at 10.0 kV. For analysis, the small fragments of the samples were mounted on silica stubs, and gold spluttered under a vacuum. An electric potential of 10.0 kV was maintained during the procedure. SEM microscopy was performed to study and characterize the uniformity of the coating.

### 2.8. Contact Angle Measurement

The 3D-printed grafts were printed for contact angle measurement, otherwise not possible with the cuttings of the marketed urinary Foley catheter because of the curved surface. The 3D CAD design of the samples was made with Shapr3D (Shapr3D, Budapest, Hungary) and the printing of the reservoirs was developed with a 3D printer (Craft-Bot Plus + Pro, Craftunique Kft., Budapest, Hungary) using fused deposition modeling (FDM) technology. Polylactic acid material was used and the samples were printed with 0.2 mm layer height with a 0.4 mm nozzle. The temperature was maintained at 210 °C for the material and 60 °C for the Kapton-coated glass build platform. 

The contact angle was measured to investigate the behavior of the 3D-printed disks in contact with the hydrophobic and hydrophilic liquids by using polar and non-polar liquids (water and diiodomethane respectively) through sessile drop method using DataPhysicsOCA20 (DataPhysics Instruments GmbH, Filderstadt, Germany) [36,37]. For this purpose, the droplet of water, 10 µL in volume, was dropped from the calibrated syringe onto the 3D-printed disks. The same process was repeated with the apolar solvent. The contact angle was then measured, during which the interfacial tension of the polar component ($\gamma_{s}^{P}$) and dispersive component ($\gamma_{s}^{d})$ of 3D-printed disks could be determined. The sum of these quantities gives the surface free energy ($\gamma^{*}$ ) of the material (Equation (2)):

(2) $$\gamma^{*}=\gamma_{s}^{d}+\gamma_{s}^{P}$$

The polarity of the 3D-printed disks can be calculated using the ratio of polar surface energy and surface free energy according to Equation (3):

(3) $$\mathrm{Polarity}\;(\%)=\frac{\gamma_{s}^{P}}{\gamma^{*}\;}\;\times \;100$$

### 2.9. Retention of the Coating on the Catheter’s Surface

The catheter was cut into fragments and coated with polymeric mixture containing phthalocyanine green (0.5 mg) to visually examine retention time. After drying the catheter pieces, they were placed in 0.05 M phosphate buffer, pH 6.8 at 37 °C on a shaker set at 30 rpm. The catheters were then visually observed between 12 and 78 h [20]. Uncoated catheters stained with an ethanolic solution of phthalocyanine green served as control to demonstrate the retention behavior in the absence of polymer coating.

### 2.10. In Vitro Release Study

Dissolution studies were carried out at 37 °C on a shaker plate at 50 rpm. The CHX-CS coated catheter pieces were immersed in 100 mL artificial urinary media pH 6.5. The sample was withdrawn at pre-determined time intervals and replaced with fresh dissolution media to maintain the sink condition. The cumulative percentage of CHX released at each time point was quantified by using a UV-visible spectrophotometer (Jasco V-730, Budapest, Hungary) at a wavelength of 255 nm. All experiments were conducted in triplicate [20]. 

### 2.11. Microbiological Studies and Biofilm Model

The developed antimicrobial coating was evaluated for its ability to inhibit the growth of three species of micro-organisms commonly associated with urinary tract infections. The bacterial strains *E. coli*, *S. aureus*, and *P. aeruginosa* were used. These bacterial strains were cultivated on blood agar plates and subjected to overnight incubation at a temperature of 37 °C. The microbial concentration was adjusted to a density of 0.5 McFarland, following the guidelines provided by the European Committee on Antimicrobial Susceptibility Testing (EUCAST) by using the disk diffusion method [38]. After 72 h, 10 µL was extracted from the interior of catheter fragments. Subsequently, the sample was diluted by a factor of one million and applied over the surface of a blood agar plate. The medium was thereafter placed in an incubator and left to incubate overnight at a temperature of 37 °C. The next day, the number of viable bacterial colonies that had grown was calculated to determine the number of living germs present in the artificial urine samples after 72 h. 

Additionally, the development of biofilms on the urinary catheter’s surface was examined. To assess biofilm formation, a crystal violet staining technique was employed [39]. In brief, the liquid supernatant was collected and the tubes and catheter fragments were gently rinsed with distilled water. Subsequently, 1% *w*/*v* crystal violet solution was added to the tubes, precisely 1 mL, to stain the cells adhered to the walls. After 25 min, the crystal violet solution was removed and the tubes were washed with distilled water. The evaluation of biofilm formation was performed by assessing the intensity of the color that was attached to the surface. The biofilm growth grading system ranged from negative (−) to strong (++) [40]. 

## 3. Results and Discussion 

### 3.1. Coating of the Catheters Following the QbD Approach

#### 3.1.1. Initial Knowledge Space Development 

**Figure 2:**
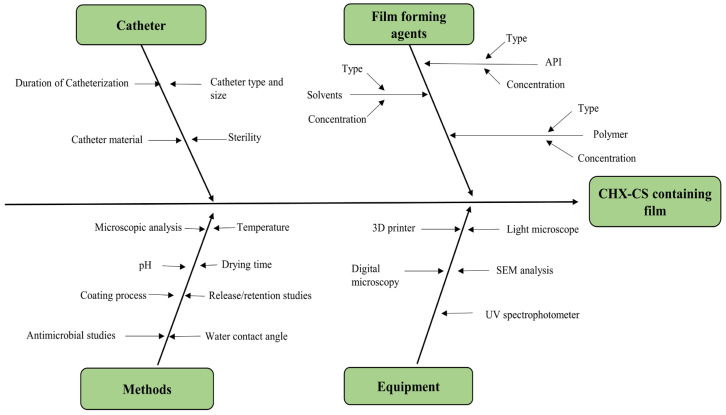
Ishikawa diagram of the target product (polymeric coating on catheters) and related factors [41,42].

In this study, we employed the commonly used Ishikawa diagram which is the development of a knowledge space as a fundamental tool for the visualization and assessment of literature. This method belongs to the category of “cause and effect” diagrams, which facilitates in the gathering of both the effects and potential causes that impact the quality of a product or process. Figure 2 represents the Ishikawa sketch for the quality development process of CHX-CS coating on catheters. Moreover, Tables S2 and S3 (Supplementary Files) enlist the range of possible QTPP elements and CQAs, respectively. Based on the QTPPs to achieve the quality, the aim was to develop the coating on the surface of the marketed Foley catheters with high uniformity and microbiological stability. The drug, CHX, must show a sustained profile to maintain the effective drug concentration at the target site to allow for long-term antibiofilm activity. Altogether, the fabrication of the optimal antimicrobial coating can improve patient compliance in a large group of the population, removing discomfort because of dryness and providing lubrication along with pharmacological action using a biocompatible polymer. 

#### 3.1.2. Risk Assessment

**Figure 3:**
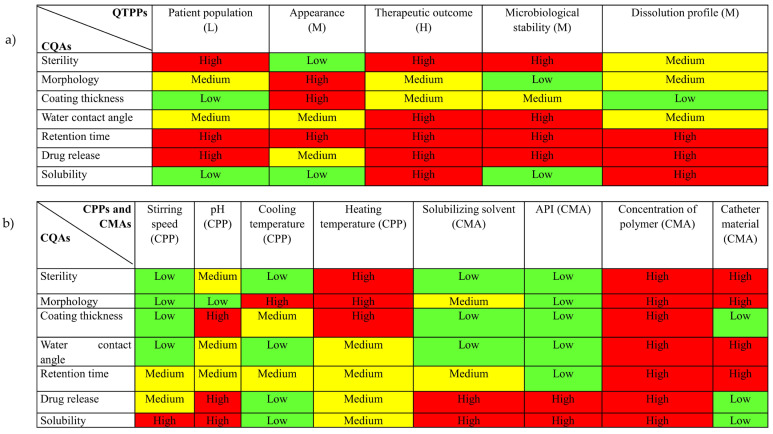
(**a**) Risk estimation matrix/interdependence rating between QTPP and CQAs, and (**b**) risk estimation matrix/interdependence rating between CPP/CMAs and CQAs.

The primary objective of risk assessment is to identify and scrutinize all the factors involved. It identifies not only the significant factors but also the more critical ones. After the determination of QTPPs, lean QbD software was employed to obtain a risk estimate matrix (REM). This is a tool that facilitates the identification of the critical quality parameters necessary for the target product by establishing a relation between different parameters. This is achieved by establishing the interdependence between the QTPPs and CQAs (Figure 3a), and CPPs/CMAs and CQAs (Figure 3b) on a three-level scale, Low, Medium, and High.

**Figure 4:**
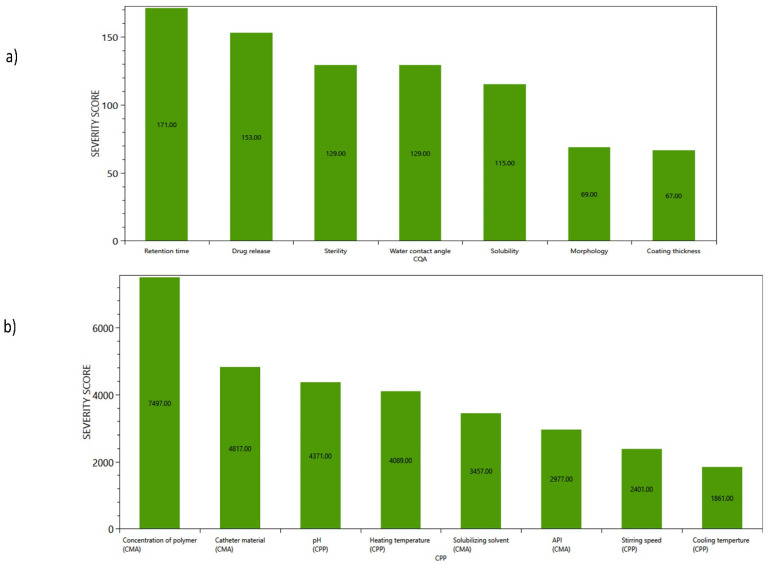
(**a**) Pareto chart presenting the severity scores based on probability rating of CQA and (**b**) Pareto chart presenting the severity scores based on the probability rating of CPPs/CMAs.

After analyzing the risk estimate matrix, Pareto charts were generated to display the severity scores as shown in Figure 4a,b. The graphs highlight the parameters that significantly impact the quality of the product during the developmental stage. The findings indicate that the coating retention time, drug release, sterility, and water contact angle are the high-risk factors, with the highest severity score. The most influencing factors responsible for the development of coating are the concentration of polymer (CS), catheter material, pH, and temperature maintained throughout the coating process.

### 3.2. Design of Experiments

After performing risk assessment by Lean QbD software^®^, the highly critical CMAs and CPPs were prioritized by using BB factorial design. Catheters with CHX-CS coating were prepared according to the 15 experimental runs generated by STATISTICA^®^12 software as shown in Table S4 (Supplementary Files) and were studied for contact angle (polarity), coating retention time, and drug release analysis.

The obtained second-degree polynomial model for the estimation of Y_1_ (polarity), Y_2_ (retention time), and Y_3_ (% drug release) is as follows:

Y_1_ = 37.04 + 13.45 X_1_ − 5.89 X_2_ − 5.22 X_3_ − 2.64 X_1_X_2_ − 5.72 X_1_X_3_ − 2.32 X_2_X_3_ + 1.16 X_1_^2^ + 2.24 X_2_^2^ + 1.05 X_3_^2^ (4)

Y_2_ = 23.08 + 12.25 X_1_ + 5.50 X_2_ − 1.25 X_3_ − 2.00 X_1_X_2_ + 7.5 X_1_X_3_ − 11 X_2_X_3_ − 3.45 X_1_^2^ + 2.29 X_2_^2^ − 2.95 X_3_^2^ (5)

Y_3_ = 71.13 − 0.75 X_1_ − 10.41 X_2_ − 1.11 X_3_ + 8.06 X_1_X_2_ + 1.27 X_1_X_3_ − 2.09 X_2_X_3_ + 0.002 X_1_^2^ + 1.10X_2_^2^ + 4.25 X_3_^2^ (6)

From the equation of Y_1_ (Equation (4))_,_ the most significant influencing factor for polarity was the concentration of CS. CS acts as a polycationic substance in an acidic environment; as the concentration of CS increases, the molecules of the chitosan become closer, resulting in high interactions between their polar group, and this can lead to the formation of a gel-like structure where polar groups interact more extensively resulting in increased polarity due to a more aligned dipole moment. Similarly, from the equation for Y_2_ (Equation (5)), again, the concentration of CS is a critical factor affecting the coating retention time due to high polymeric adhesion. However, from the equation for Y_3_ (Equation (6)), the most critical factor was found to be the pH of the sample. As the pH increased, drug release decreased. The slower release rate of CHX from the surface of the coated urinary catheter is primarily due to the moderate solubility of protonated CHX at this pH (4.8–5.0). The moderate solubility can result in a slower release rate compared to conditions where urinary pH is either more acidic or more alkaline. Another responsible factor can be interaction with catheter material. The interaction between the catheter material and CHX can also vary with the change in pH, as there might be stronger adsorption which will slow down the diffusion rate from the catheter surface to the surrounding media.

The Pareto charts as shown in Figure S1a–c (Supplementary Files) also established similar outcomes with the probability of the impact of critical parameters on the dependent variables (polarity, retention time, and drug release) individually. A *t*-test was also performed to determine the significance indicated by the vertical line passing through the variables. The concentration of CS was the most influential material attribute with a direct impact on the polarity and the retention time of the coating on the catheter’s surface, whereas pH was found to be the CPP most directly affecting the % drug release from catheters in the artificial urinary media. 

Also, the 3D surface plots in Figure S2 (Supplementary Files) were obtained from the software which shows the impact of highly critical independent variables on the dependent variables graphically. Figure S2a–c present the 3D plots showing the effect of the concentration of chitosan (X_1_) and pH (X_2_) on the polarity, coating retention time, and drug release from CHX-CS coatings. Polarity and coating retention time increase with the increase in concentration of CS. However, the drug release rate decreases with an increase in the concentration of CS. 

Additionally, the contour plots in Figure S3 (Supplementary Files) were obtained which show the relation between the two independent variables and the dependent variable. Figure S3a–c show the contour plots for the effect of the independent variables on the % polarity, coating retention time, and drug release. The polarity and coating retention time increase with the increase in concentration of CS and pH. However, the drug release rate decreases with an increase in the concentration of CS and a decrease in pH. Altogether, the polynomial equations, Pareto charts, surface plots, and contour plots present the analogous findings.

### 3.3. Weight-Gain Analysis of Coated Catheter 

The weight gain of the catheter samples was evaluated by weighing them before and after the coating as shown in Table S5 (Supplementary Files) to determine the increase in weight, which ranged from 1 to 2.2 mg. The weight gain shows that the coating is present on the surface of the urinary catheters. 

### 3.4. Morphological Analysis Using SEM

**Figure 5:**
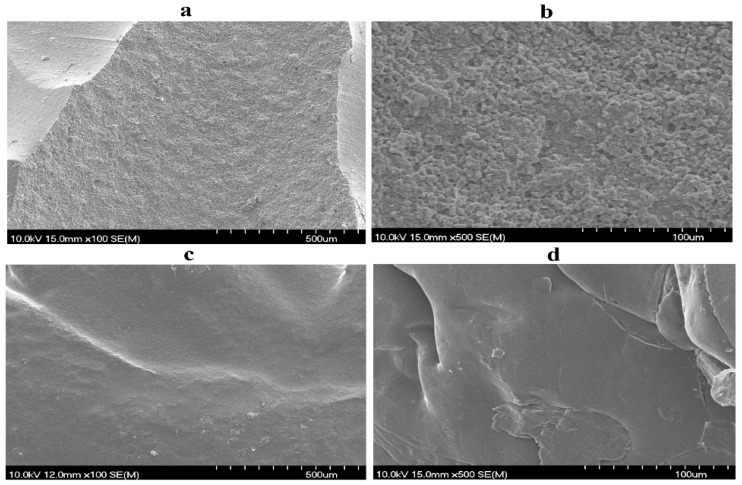
SEM micrographs; (**a**,**b**) are for the uncoated catheter samples at a scale of 500 and 100 microns, respectively, and (**c**,**d**) show the polymeric coated samples at a scale of 500 and 100 microns, respectively.

SEM images of the coated and uncoated samples were taken as shown in Figure 5, and the difference between the uncoated and coated catheter samples was studied. The SEM images of the coated catheters present a smooth and homogenous morphology in comparison to uncoated samples showing roughness and ridges. The micrographs indicate the successful application of the coating on the catheter surface. The successful and strong adhesion of the chlorhexidine and chitosan coating on the surface of silicone Foley catheters can be explained by several factors related to the materials and methods used in this study. The cationic nature of chitosan facilitates electrostatic interactions with the silicone surface, enhancing the adhesiveness of coating. These electrostatic forces are supplemented by hydrogen bonding and Van der Waals forces which further stabilize the coating on the catheter [43]. In addition, the rough surface of uncoated catheters increases the available surface area, encouraging mechanical interlocking resulting in a stronger physical grip of the coating. Also, the repeated cycles of dip coating gradually build up layers, ensuring both uniformity and robustness in adhesion. The slightly acidic pH during the preparation of the coating enhances the solubility of chitosan, optimizing its affinity for the silicone surface and allowing more effective bonding, especially in the presence of chlorhexidine [44]. 

### 3.5. Contact Angle Measurement 

The contact angle measurement of the coated and uncoated 3D-printed disks presents different degrees of polarity as shown in Table S3 (Supplementary Files). According to the different contact angles (as shown in Table S6 Supplementary Files) and polarity, these samples are categorized into three different groups:

Low polarity (<35%): Samples 1, 3, 5, and 12 show low contact angles and polarity, which indicates that they exhibit higher wettability properties and great adhesion of the coating on the 3D-printed disks. However, low contact angles and polarity also indicate that the catheter surface will be highly susceptible to biofilm formation because of a higher surface energy. 

Moderate polarity (35–45%): Samples 4, 7, 8, 9, 10, 11, 14, and 15 show moderate contact angles and polarity implicating that they exhibit a good balance between wettability properties and hydrophobicity, which is favorable for biomedical applications, as they provide sufficient biocompatibility and prevent biofilm formation as compared to low-contact-angle samples. 

High polarity (>45%): Samples 2, 6, and 13 show high contact angles and polarities, leading to poor wettability properties. While high contact angles or polarity may reduce the biofilm formation and increase the antimicrobial efficacy, they may affect the adhesion of the coating on the 3D-printed surface. Moreover, the higher polarity also enhances the coating’s efficacy in preventing biofilm formation by improving its antimicrobial properties through better interactions with bacterial cell membranes. 

C8 shows a higher contact angle with water when compared to C2 while maintaining a good level of wettability. The lower contact angle with diiodomethane and moderate polarity indicates that the catheter surface will effectively prevent the bacterial adhesion essential for reducing catheter-associated infections. To conclude, out of all the samples, C2 (water: 31.94 degrees, diiodomethane: 34.84 degrees, polarity: 48.21%) and sample 8 (water: 57.13 degrees, diiodomethane: 28.69 degrees, polarity: 38.47%) are the optimal samples.

### 3.6. Coating Retention Analysis

The coating retention study was carried out for the catheter samples based on experimental runs obtained through DoE. The retention time for all the samples varied considerably indicating the influence of the different experimental conditions as shown in Table S3 (Supplementary Files). Specifically, samples C2 and C8 showed the longest retention time of 27 h and 37 h, respectively, facilitating the sustained release of CHX for improved antimicrobial activity. Sample C2 (2.5% CS, pH 4.8, and temperature 30 °C) and sample 8 (2.5% CS, pH 5.0, and temperature 40 °C) show an enhanced retention time as compared to other samples because of their high chitosan concentration and temperature. The higher concentration of CS results in a denser and stable coating matrix reducing the rate of drug release. The high temperature also promotes effective coating because of the high gelation rate.

### 3.7. In Vitro Release Study

**Figure 6:**
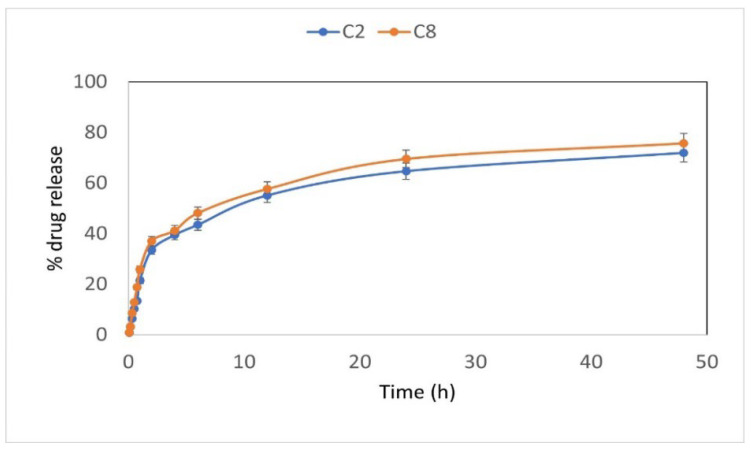
Release profile of CHX from different formulations C2 and C8.

The coated catheters developed based on the experimental runs generated by BB design with different concentrations of CS, pH, and temperature were studied for drug release (Figure 6). The drug release profile for all 15 samples demonstrated different % release of CHX, as shown in Table S3 (Supplementary Files). The highest drug release of 86.56% was presented by sample C1 composed of 1.5% *w*/*v* of CS indicating that a low concentration of polymer promotes high drug release. On the contrary, higher polymer concentration slows down the % release of the drug which correlates well to achieving a sustained release pattern. Peculiarly, sample C12, with 2.0% *w*/*v* of CS, showed the lowest drug release of 63.26% as compared to the coatings with 2.5% *w*/*v* of CS.

### 3.8. Microbiological Studies 

**Figure 7:**
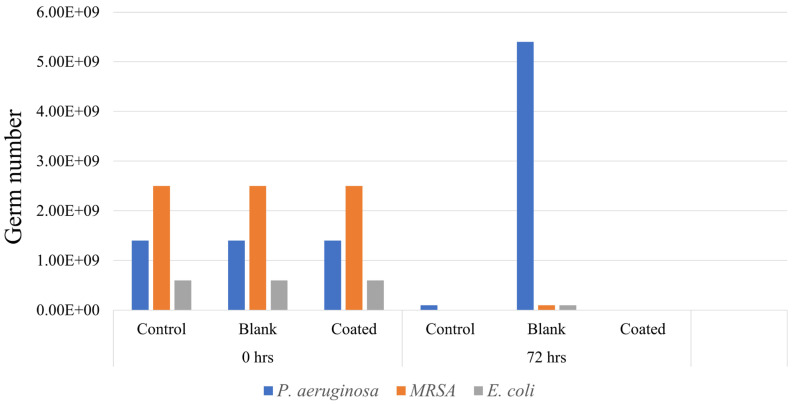
No. of germ count at 0 h and at 72 h for all three bacterial strains with control (chitosan coated), blank (uncoated), and coated (CHX-XS coated) catheter samples.

The primary cause of UTIs is the adhesion of micro-organisms on the surface of urinary catheter, which is subsequently followed by biofilm formation. This was reported in patients with bladder dysfunction or using indwelling urinary catheters. A promising approach to prevent bacterial adhesion on the catheter surface involves the application of an antimicrobial coating with controlled drug release properties on the surface of the catheter. In this study, we presented the successful coating of chlorhexidine and chitosan gel on the catheter surface. Formulations C2 and C8 were chosen for the microbiological studies because of their better experimental outcomes. Microbiological assessment was performed by adjusting the microbial concentration to 0.5 McFarland and incubating bacterial strains on blood agar plates at 37 °C for 72 h. After incubation, viable bacterial colonies were counted to determine the no. of germs present in the artificial urine sample where blank represents the catheter without any coating, control represents the catheter with chitosan, and coated represents the catheter coated with chlorhexidine and chitosan gel. Figure 7 demonstrates that the coated catheter samples showed significant reduction in all three bacterial strains in 72 h as compared to the blank and control samples indicating the potential of the coating to mitigate the risk of UTIs. In the case of the control samples, *S. aureus* and *E. coli* strains have been significantly inhibited, whereas *P. aeruginosa* strains are still present even after 72 h. The results demonstrated that the catheter coating effectively inhibits the growth of bacteria when enough chlorhexidine is present in the coating. Chitosan all alone can inhibit the growth of *S. aureus* and *E. coli*, but it could not inhibit the growth of *P. aeruginosa* completely.

**Figure 8:**
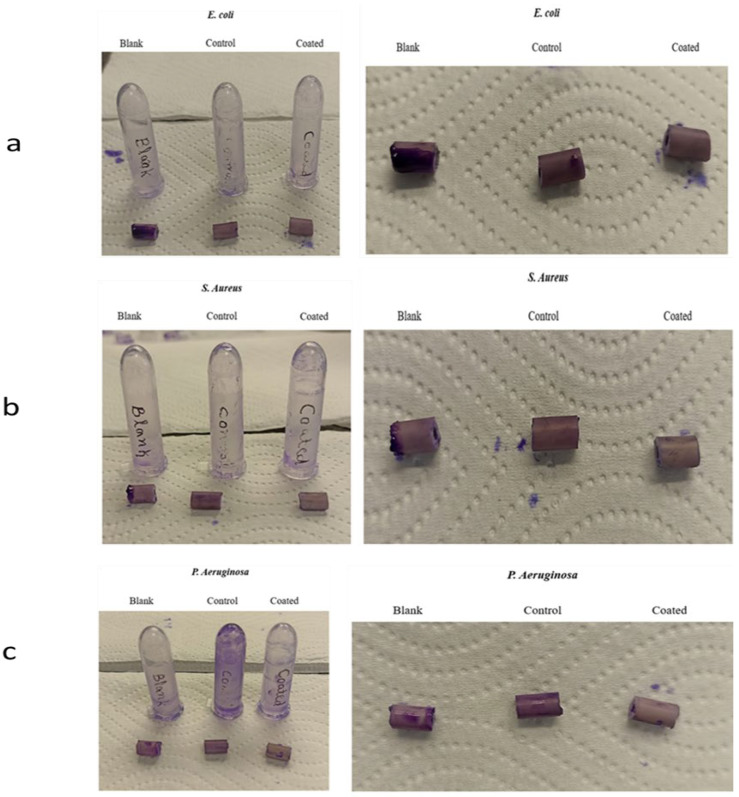
Biofilm formation on the surface of urinary catheter pieces with *E. coli* strain (**a**), *S. aureus* (**b**), and *P. aeruginosa* (**c**), where control represents catheter with chitosan, blank represents uncoated catheter, and coated represents catheter coated with CHX-CS gel.

Additionally, a visual examination of the biofilm formation using the crystal violet dye confirmed these findings. Figure 8a–c shows that the blank catheter displayed the most intense staining, followed by the control samples and coated samples of the catheter with all the three bacterial strains. The results demonstrate that the incorporation of chlorhexidine with chitosan in the coating resulted in the almost complete prevention of biofilm formation, whereas the blank sample exhibited a high tendency to support biofilm growth, and the control samples demonstrated some tendency to support biofilm growth by *P. aeruginosa.* This indicates that the controlled release property of the coating enabled an optimal CHX-CS release rate to inhibit the bacterial adhesion and biofilm formation on the surface of the catheter. Hence, these findings prove the potential of chlorhexidine–chitosan gel coatings in reducing bacterial colonization. 

## 4. Conclusions

In this study, the risks associated with CAUTIs were taken into consideration to improve patient compliance with intravesical drug delivery. Urinary catheters are widely used medical devices across the geriatric population and nearly half of patients suffer from CAUTIs. Keeping this in mind, an atypical strategy was developed which not only prevented the formation of biofilms in the inserted catheters and therefore infections, but also acted as a reservoir for the sustained delivery of CHX to reduce the occurrence of CAUTIs. For this purpose, we employed a holistic risk and science-focused QbD approach to establish the critical process and material characteristics that may influence the quality of the desired product, herein, the antimicrobial CHX-containing polymeric-coated urinary catheter. Moreover, the use of DoE proposed a practical approach for reducing the trial costs and standardizing the coating process by limiting errors. A simple cost-effective dip-coating technique was used to coat the catheters with the CS and CHX mixture. The coatings retained on the surface of the catheters for a long duration provided sustained drug release and demonstrated optimal polarity and surface wettability. The most optimized samples with the desired outcomes were then selected for further analysis through microbiological studies. CHX-CS coating presented promising antibiofilm properties along with the reduction in the count of bacteria adhering to the catheter’s surface, contributing to the prevention of colonization. The reported outcomes from this study suggest possible clinical intervention in the geriatric population. All in all, this rationale can be easily commercialized because of the low production cost and easy approach. However, further investigations such as animal studies are needed to establish the in vivo efficacy and therapeutics.

## Figures and Tables

**Table 1:** Summary of chlorhexidine and chitosan gel coating preparation and application process on Foley catheters [24].

S. No.	Steps	Details
1.	Chitosan stock solution preparation	Concentrations: 1.5%, 2.0%, and 2.5% Polymer dissolved in 0.05 M acetic acid using magnetic stirrer pH maintained between 4.8 and 5.2 using 0.1 N NaoH
2.	Preparation of CHX coating solution	CHX (1%) solubilized in chitosan solution
3.	Catheter preparation	Silicone Foley catheters (14 Fr, 4mm diameter) Cut into 1.5 cm long pieces Cleaned with water and ethanol mixture Air dried before coating
4.	Dip-coating process	Catheters dipped in CHX-CS gel coating Air dried to form a monolayer coating Process repeated until uniform coating was achieved
5.	Temperature during coating	Coating mixture was heated at 20 °C, 30 °C, and 40 °C in different runs until clear polymeric mixtures were obtained
6.	Post-coating procedure	Coated catheters were air dried and then studied for weight analysis to confirm the coating thickness

## Data Availability

The original contributions presented in the study are included in the article/Supplementary Materials; further inquiries can be directed to the corresponding author/s.

## Supplementary Materials

The following supporting information can be downloaded at: https://www.mdpi.com/article/10.3390/biomedicines12092032/s1, Table S1: Experimental runs generated by STATISTICA^®^ 12 software for the development of catheter coating; Table S2: Quality target product profile (QTPP) elements, their target, and justification; Table S3: List of the critical quality attributes (CQAs) of the coated catheters with their target and justification; Table S4: Experimental runs generated by STATISTICA®12 with the reported outcome on the dependent variables; Table S5: Weight gain of coated catheters, prepared by following the experimental runs using DoE; Table S6: Contact angle measurement of 15 samples with water and diiodomethane; Figure S1: Pareto charts of the effect of examined independent variables (concentration of chitosan, pH, and temperature) on the outcomes (polarity, coating retention time, and drug release ) of CHX-CS coating as (a,b,c). Bars that exceed the vertical line indicate that the terms are significant (p < 0.05); Figure S2: Surface response plots highlighting the influence of some formulation variables (concentration of chitosan, pH, and temperature) on the polarity, coating retention time, and drug release of CHX-CS coating as a,b,c; Figure S3: The contour plots presenting the influence of independent variables on % polarity (a), retention time (b), and % drug release (c).

## Abbreviations

Catheter-associated urinary tract infections (CAUTIs); Quality by Design (QbD); Quality target product profile (QTPP); Critical quality attributes (CQAs); Critical material attributes (CMAs); Critical process parameters (CPPs); Design of experiments (DoE); Box–Behnken (BB); Risk estimate matrix (REM); Active pharmaceutical ingredient (API); Chitosan (CS); Chlorhexidine (CHX); Scanning electron microscopy (SEM); Optical contact angle (OCA); The International Council on Harmonization of Technical Requirements for Pharmaceuticals for Human Use (ICH); French (Fr).
