# Supplementary material for: Investigation of Chlorhexidine and Chitosan Gel-Based Coatings for the Prevention of Intravascular Catheter-Associated Infections Following Quality by Design Approach
Source: Biomedicines. 2024 Sep 5;12(9):2032. doi: 10.3390/biomedicines12092032 (PMC11429402; doi:10.3390/biomedicines12092032)
Supplement: Supplementary file 1 [file biomedicines-12-02032-s001.zip › biomedicines-3171747-supplementary.pdf]

Supplementary files

Table S1: Experimental runs generated by STATISTICA® 12 software for the development of catheter coating

Experimental runs	Chitosan %	pH	Temperature (Celsius)
C1	1.5	4.8	30
C2	2.5	4.8	30
C3	1.5	5.2	30
C4	2.5	5.2	30
C5	1.5	5.0	20
C6	2.5	5.0	20
C7	1.5	5.0	40
C8	2.5	5.0	40
C9	2.0	4.8	20
C10	2.0	5.2	20
C11	2.0	4.8	40
C12	2.0	5.2	40
C13	2.0	5.0	30
C14	2.0	5.0	30
C15	2.0	5.0	30

Table S2: Quality target product profile (QTPP) elements, their target, and justification

QTPP elements	Target	Justification
**Route of administration**	Intravesical	The intravesical route provides an opportunity to deliver rug at the target site through a catheter to maximize effective drug concentration while minimizing the systemic side effects.
**Dosage form**	Gel coating on the catheter	To increase the residence time of the drug by the controlled/sustained release at the site of action.
**Site of action**	Urinary tract	For long-term local therapy with urinary catheters
**Drug classification/target**	Chlorhexidine	Because of its antibacterial properties and high safety profile.
**Gelling polymer**	Chitosan	Because of its high compatibility and antibacterial properties, ability to form a gel with high

		concentration and heating along with its biodegradation.
**Therapeutic outcome**	Local	The intravesical route can be used for local drug delivery.
**Physical appearance of the gel**	Transparent and homogeneous	Necessary for the smooth and uniform coating with optimal thickness.
**Dissolution profile**	Sustained release	The release of the drug should be sustained to achieve long-term therapy to avoid the frequent catheter insertion, causing wear and tear along with inflammation and unease.
**Packaging**	Sealed well and easy to open to avoid unnecessary touching to the surface	To avoid microbial contamination.
**Microbiological stability of drug**	No contamination of the prepared stock along with its ability to maintain the bacteriostatic or bactericidal activity.	CHX is stable in the solution form and therefore available as mouthwash in the market.
**Indication (Patient population)**	For pediatrics, adults, and geriatrics with chronic and acute urinary tract issues	Commonly used for urinary tract problems.

Table S3: List of the critical quality attributes (CQAs) of the coated catheters with their target and justification

CQA	Target	Justification
**Water contact angle or wettability**	Should be hydrophilic	Hydrophilicity allows for the smooth insertion and removal of the catheters without the need for lubricant.
**Drying time of coating gel**	Low possible time	It allows the multilayered coating and saves time.
**Solubility of drug**	Easily soluble in the gelling system	CHX is soluble in the solubilizing media of the polymer, i.e. 0.05 M GAA, thereby minimizing the time required for complete homogenous dispersion of the drug in the polymeric mixture.

**Morphology**	Homogeneous, clear, and odorless	For coating uniformity.
**Sterility**	Meet the requirements of the sterile preparations as per European pharmacopeia	To ensure microbiological activity during the insertion period.
**Retention time of coating**	High adhesion to the catheter	To ensure the coating has a prolonged adhesion to the surface of the catheter to achieve antibiofilm property.
**Drug Release**	Sustained	To maintain the constant level of drug at the target site to achieve high pharmacological activity.
**Coating thickness**	Uniform	To achieve the therapeutic outcome.
**Coating technique**	Dip coating	To ensure a uniform layer of coating on the surface of the catheter (28,29).

Table S4: Experimental runs generated by STATISTICA®12 with the reported outcome on the dependent variables

**Experimental runs (Sample no.)**	**Chitosan %**	**pH**	**Temperature (Celsius)**	**Polarity(%)**	**Retention time (h)**	**Drug release (%)**
**C1**	1.5	4.8	30	27.35	13	86.56
**C2**	2.5	4.8	30	48.21	27	71.87
**C3**	1.5	5.2	30	27.64	18	67.27
**C4**	2.5	5.2	30	43.22	28	68.71
**C5**	1.5	5.0	20	31.40	24	69.40
**C6**	2.5	5.0	20	45.81	29	73.25
**C7**	1.5	5.0	40	35.51	17	66.38
**C8**	2.5	5.0	40	38.47	37	72.78
**C9**	2.0	4.8	20	44.69	13	73.35
**C10**	2.0	5.2	20	37.57	32	65.84
**C11**	2.0	4.8	40	38.19	21	74.96
**C12**	2.0	5.2	40	26.42	18	63.26
**C13**	2.0	5.0	30	47.53	20	78.20
**C14**	2.0	5.0	30	35.47	21	73.14
**C15**	2.0	5.0	30	37.06	20	72.79

Table S5: Weight gain of coated catheters, prepared by following the experimental runs using DoE

Sample no.	Weight before coating (mg)	Weight after coating (mg)	Weight gain (mg)
1	210	211	1
2	225	227.1	2.1
3	168	169.1	1.1
4	192	194	2
5	190	192.1	2.1
6	192	193.2	1.2
7	146	147	1
8	211	212.5	1.5
9	174	176.2	2.2
10	193	194.7	1.7
11	187	188.9	1.9
12	174	175.6	1.6
13	201	203.2	2.2
14	188	189.4	1.4
15	181	182.7	1.7

Table S6: Contact angle measurement of 15 samples with water and diiodomethane

Sample no.	Water Contact angle (degree)	Diiodomethane Contact angle (degree)
1.	31.28 °	31.94 °
2.	31.94 °	34.84 °
3.	30.76 °	34.55 °
4.	37.46 °	34.55 °
5.	48.10 °	34.91 °
6.	69.24 °	47.39 °
7.	67.23 °	33.44 °
8.	57.13 °	28.69 °
9.	81.28 °	60.99 °
10.	51.15 °	30.87 °
11.	27.13 °	35.54 °
12.	30.09 °	38.33 °
13.	34.65 °	43.55 °
14.	27.86 °	36.82 °
15.	53.81 °	39.29 °

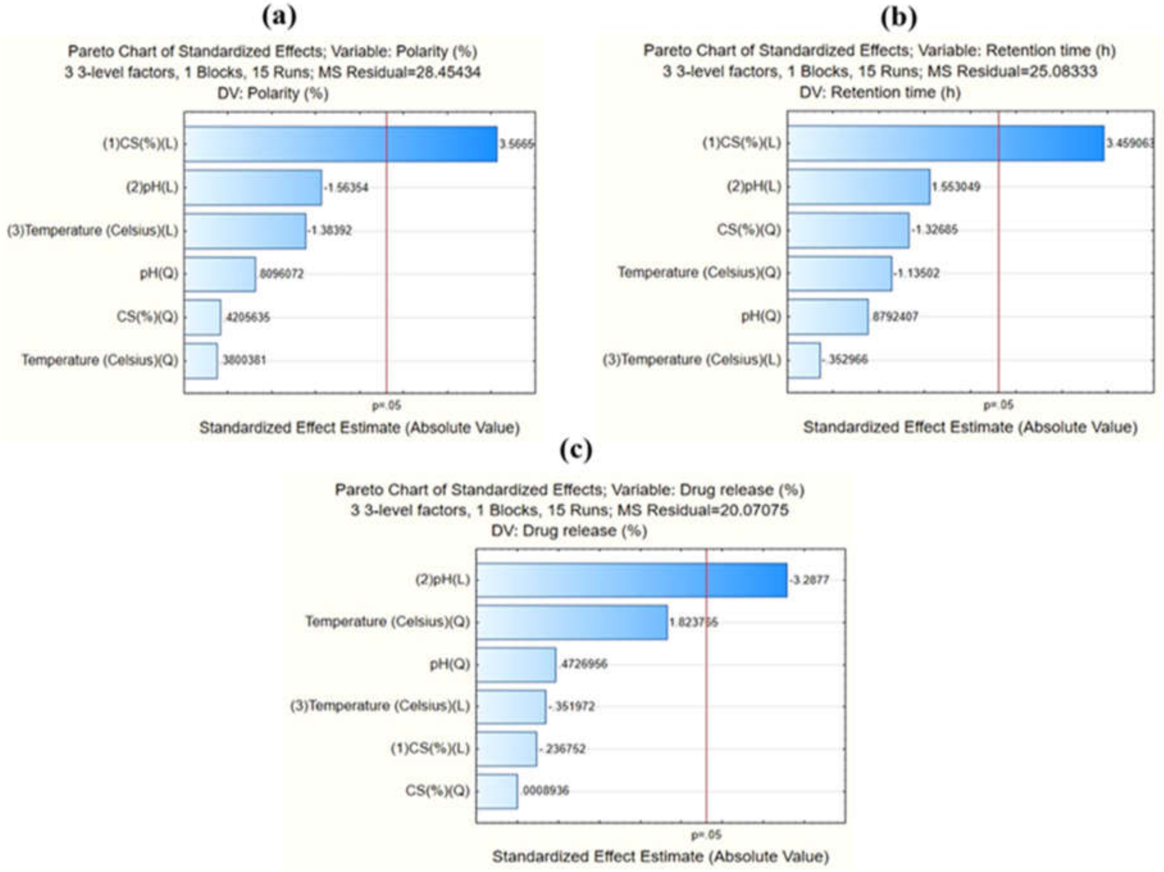

Figure S1: Pareto charts of the effect of examined independent variables (concentration of chitosan, pH, and temperature) on the outcomes (polarity, coating retention time, and drug release) of CHX-CS coating as (a,b,c). Bars that exceed the vertical line indicate that the terms are significant ($p < 0.05$).

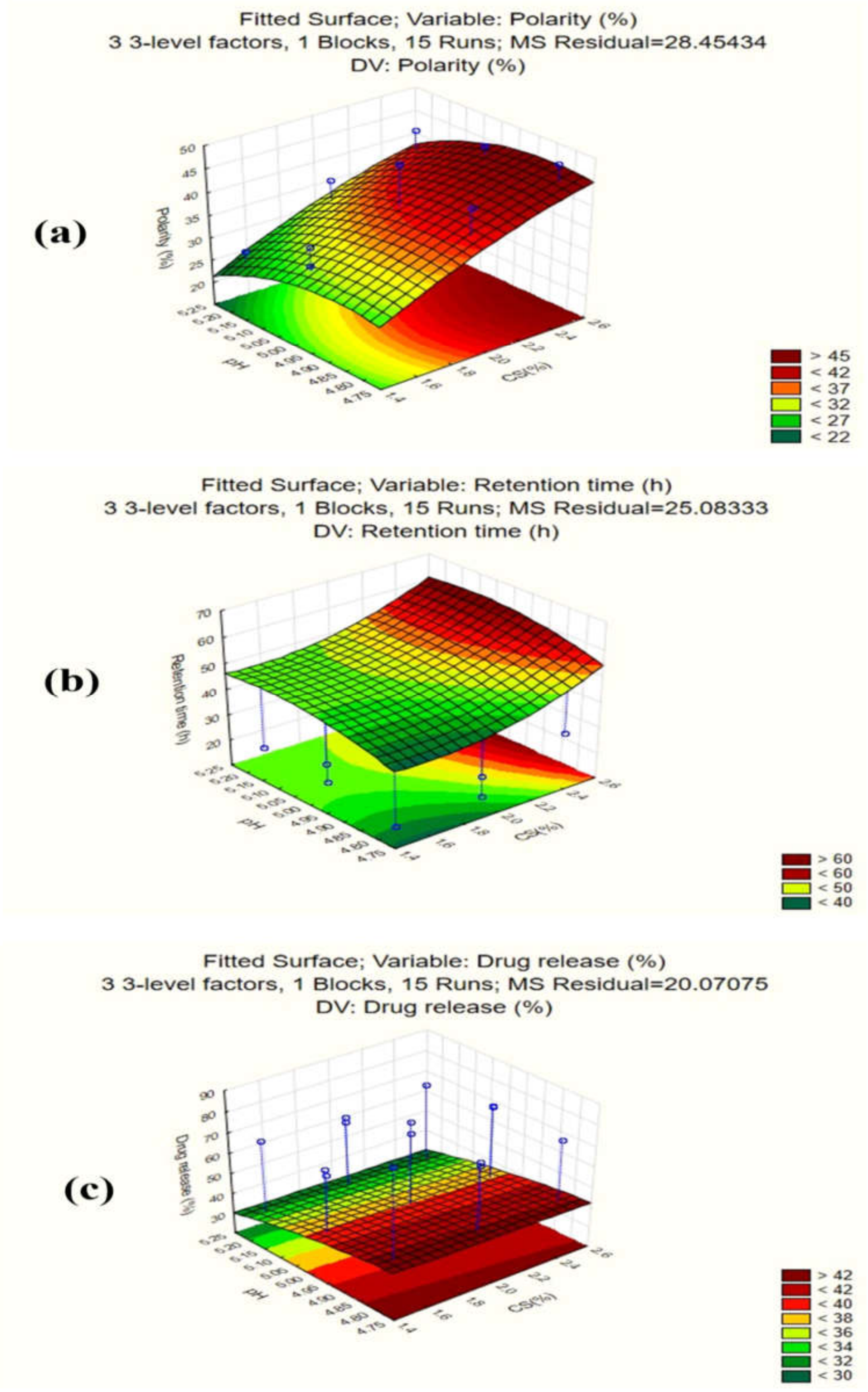

Figure S2: Surface response plots highlighting the influence of some formulation variables (concentration of chitosan, pH, and temperature) on the polarity, coating retention time, and drug release of CHX-CS coating as a,b,c.

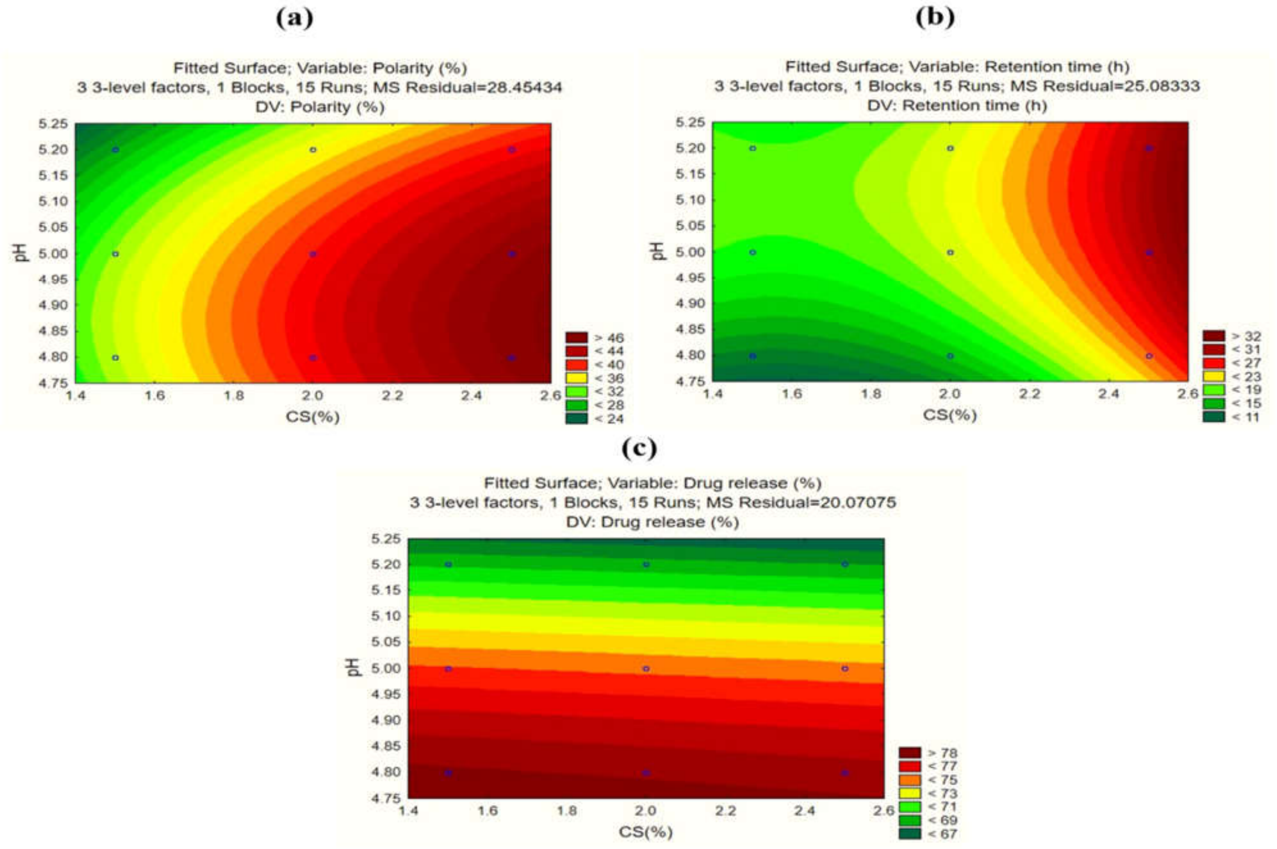

Figure S3: The contour plots presenting the influence of independent variables on % polarity (a), retention time (b), and % drug release (c).
